# Surface Phosphorus‐Induced CoO Coupling to Monolithic Carbon for Efficient Air Electrode of Quasi‐Solid‐State Zn–Air Batteries

**DOI:** 10.1002/advs.202101314

**Published:** 2021-08-08

**Authors:** Huan Liu, Yanyan Liu, Sehrish Mehdi, Xianli Wu, Tao Liu, Benji Zhou, Pengxiang Zhang, Jianchun Jiang, Baojun Li

**Affiliations:** ^1^ College of Chemistry Zhengzhou University 100 Science Road Zhengzhou 450001 P. R. China; ^2^ Institute of Chemical Industry of Forest Products CAF National Engineering Lab for Biomass Chemical Utilization Key and Open Lab on Forest Chemical Engineering SFA 16 Suojinwucun Nanjing 210042 P. R. China; ^3^ College of Science Henan Agricultural University Zhengzhou Henan 450002 P. R. China; ^4^ CAS Key Laboratory for Biomedical Effects of Nanomaterials and Nanosafety National Center for Nanoscience and Technology Beijing 100190 P. R. China

**Keywords:** bifunctional oxygen catalysts, biomass, monolithic electrodes, surface phosphorus‐induced activity, Zn–air batteries

## Abstract

One challenge facing the development of air electrodes for Zn–air batteries (ZABs) is the embedment of active sites into carbon, which requires cracks and blends between powder and membrane and results in low energy efficiency during manufacturing and utilization. Herein, a surface phosphorization‐monolithic strategy is proposed to embed CoO nanoparticles into paulownia carbon plate (P–CoO@PWC) as monolithic electrodes. Benefiting from the retention of natural transport channels, P–CoO@PWC‐2 is conducive to the construction of three‐phase interface structure for efficient mass transfer and high electrical conductivity. The electrode exhibits remarkable catalytic activities for both oxygen reduction reaction (ORR) and oxygen evolution reaction (OER) with a small overpotential gap (*E*_OER_ − *E*_ORR_ = 0.68 V). Density functional theory calculations reveal that the incorporation of P on P–CoO@PWC‐2 surface adjusts the electronic structure to promote the dissociation of water and the activation of oxygen, thus inducing catalytic activity. The monolithic P–CoO@PWC‐2 electrode for quasi‐solid‐state or aqueous ZABs has excellent specific power, low charge–discharge voltage gap (0.83 V), and long‐term cycling stability (over 700 cycles). This work serves as a new avenue for transforming abundant biomass into high‐value energy‐related engineering products.

## Graphical abstract

Carbon electrodes manufactured from wood by a surface phosphorization‐monolithic strategy exhibit outstanding bifunctional activities in Zn–air battery. The surface P atoms promote the dissociation of water and the activation of oxygen, thus inducing efficient catalytic activity.

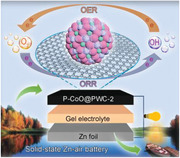

## 1 Introduction

With the increasing energy crisis and serious environmental pollution caused by the use of fossil fuels, researchers are committed to making efficient use of renewable energy by developing conversion and energy storage technologies.^[^ ^1^, ^2^ ^]^ Energy conversion technology based on a rechargeable Zn–air battery (RZAB) has been increasingly studied because of its high theoretical energy density, high safety, and low manufacturing cost.^[^ ^3^, ^4^ ^]^ Up to date, the energy conversion efficiency of this technology is limited by the slow oxygen reduction reaction/oxygen evolution reaction (ORR/OER) on the air electrode.^[^ ^5^, ^6^, ^7^ ^]^ Recent landmark catalysts are platinum‐ and iridium/ruthenium‐based catalytic materials. Unfortunately, the poor bifunctionality and the high cost of these precious metal‐based catalysts severely hinder their wide application.^[^ ^8^, ^9^, ^10^ ^]^ Hence, the rational design of highly active and low‐cost bifunctional oxygen catalysts remains a challenging and imperative problem.

Over the past decades, biomass‐derived carbon materials have been considered ideal substrates for electrocatalysts because of their renewable, eco‐friendly, and low‐cost advantages.^[^ ^11^, ^12^, ^13^ ^]^ To date, biomass carbon materials have been tentatively studied for energy‐related applications. Mostly, biomaterials are first broken down into molecular precursors and then reassembled into doped toners. This preparation route inevitably leads to an increase in interfacial resistance, the blockage of catalytic active sites, and the mechanical exfoliation of catalysts from electrodes, all of which severely decrease catalytic efficiency and the durability of catalysts.^[^ ^14^, ^15^, ^16^, ^17^ ^]^ Wood microfibers possess an abundantly porous hierarchical structure.^[^ ^18^ ^]^ The natural porous channels in plants for transporting nutrients (water, oxygen, organics, and ions) to ensure growth are beneficial to fabricate effective three‐phase interfaces (air–electrolyte–catalyst) in RZABs.^[^ ^19^, ^20^ ^]^ Thus, it is highly promising to use the natural porosity of wood to create channels and produce carbon plates embedded with catalytic active sites. This energy‐saving and simple method facilitates catalysis reactions at the three‐phase interface and improves electrical conductivity. There have been inspiring reports on nonprecious metals operating as catalytic active sites and embedded into biomass carbon to produce monolithic porous electrodes for oxygen‐involved reactions.^[^ ^18^, ^21^ ^]^

The activities of the ORR and OER are inhibited by the sluggish activation of water and oxygen due to the stable surface electronic properties of inert biomass carbon materials. After doping with transition metal oxides (TMOs), carbides, and nonmetals (such as N, P, S, and B), the ORR and OER activities are enhanced due to the tuning effect triggered by the electronegativity and atomic size of the dopants.^[^ ^22^, ^23^, ^24^ ^]^ In particular, Co‐based oxides (CoO*_x_*) have been studied as ORR catalysts, due to being abundant and easy‐to‐prepare. Efficient hydroxyl desorption behavior on oxide surfaces is demonstrated to be effective in inducing to excellent OER performance.^[^ ^25^, ^26^, ^27^, ^28^ ^]^ To meet the bifunctional performance requirements of the ORR and OER, heteroatoms are introduced to adjust the electronic structure and to promote the oxygen activation ability. In particular, P atoms promote the transfer of electrons around P to O, because P has a much lower electronegativity than O and the P—O bond is highly polarized. Theoretically, surface P atoms act as an electron transfer bridge to improve the overall conductivity, cooperatively improving the oxygen activation ability of CoO*_x_*. However, there is presently no successful case that involves such a surface design strategy to consider both of the catalytic activity and environmental benign.^[^ ^29^, ^30^ ^]^

Based on the above theoretical analysis, this research reports a monolithic catalytically active carbon electrode consisting of surface phosphorus‐induced CoO particles coupled with biomass carbon plates (P–CoO@PWC). P–CoO@PWC‐2 exhibits remarkable Pt‐like ORR activity (onset/half‐wave potential of 0.91/0.84 V) and high OER activity (overpotential of 290 mV at 10 mA cm^−2^). The key to the improved activities is attributed to the effective activation of water and oxygen molecules induced by surface doping P atoms into CoO@PWC. Surface phosphorus‐induced activity is contributed by a large abundance of defect sites and the changed coordination environment around the metal species. The partial activation between the P atoms and CoO provides the metal defects with robust stability. This monolithic electrode for quasi‐solid‐state or aqueous ZABs displays excellent specific power, a low charge–discharge voltage gap (0.83 V) and long‐term cycling stability (over 700 cycles). Therefore, this surface phosphorization‐monolithic strategy for electronic modulation demonstrates a new rational design of efficient electrocatalysts for use in energy conversion facilities.

## 2 Results and Discussion

### 2.1 Fabrication and Morphology

**Figure 1:**
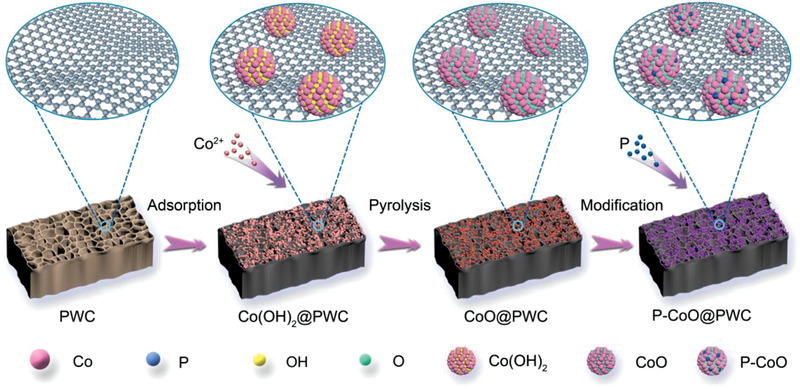
Schematic showing the procedure for synthesizing P–CoO@PWC.

**Figure** **1** displays the schematic for preparing the catalytically active carbon electrode. The whole formation process of each electrode took place on a visual macroscopic 3D porous carbon plate. First, raw paulownia wood was carbonized to remove hydrogen and oxygen, thereby improving the electrical conductivity of the produced carbon. Then, Co(OH)_2_ was uniformly grown in a macroporous channel by hydrothermal precipitation method. Finally, CoO@PWC was readily fabricated via pyrolysis. In the subsequent surface‐phosphorization process, NaH_2_PO_2_ was used as a P precursor due to its low toxicity. NaH_2_PO_2_ dissociated to form PH_3_ gas after heating to phosphorize the CoO@PWC surface. The phosphorization was extended to proceed the extent of phosphorization and improve the P content in the resultant P–CoO@PWC. The P–CoO@PWC family of electrocatalysts, which were obtained with different phosphorization durations, are designated P–CoO@PWC‐1, P–CoO@PWC‐2, and P–CoO@PWC‐3, corresponding to phosphorization of 15, 30, and 60 min, respectively.

**Figure 2:**
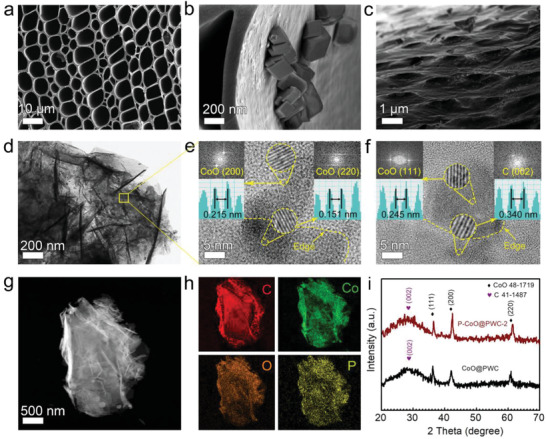
a–c) SEM images of PWC, CoO@PWC, and P–CoO@PWC‐2. d) TEM image of P–CoO@PWC‐2. e,f) HRTEM images of P–CoO@PWC‐2 (including the corresponding FFT patterns and the atomic intensity profiles). (g,h) EDX‐STEM elemental mappings of P–CoO@PWC‐2. i) XRD patterns of CoO@PWC and P–CoO@PWC‐2.

The morphology and surface structure of PWC, CoO@PWC, and P–CoO@PWC‐2 were observed by scanning electron microscopy (SEM). After pyrolysis, the anisotropic 3D structure of PWC is well preserved with long channels in the vertical direction and well‐arranged pores in the parallel direction (**Figure** **2**a). This macroporous structure provides sufficient channels and space for the formation of CoO. Thus, the resulting CoO@PWC (Figure 2b) and P–CoO@PWC‐2 (Figure 2c) inherit the overall flake morphology of the precursor Co(OH)_2_@PWC (Figure S1, Supporting Information). Due to the dehydration and gas release of CoO@PWC during the phosphorization process, the surface of P–CoO@PWC‐2 becomes porous and rough. This porous structure was confirmed by N_2_ adsorption–desorption experiments (Figure S2, Supporting Information). The specific surface areas of PWC, CoO@PWC, and P–CoO@PWC‐2 are measured to be 355, 626, and 985 m^2^ g^−1^, respectively. Compared to PWC and CoO@PWC, the significantly enhanced pore size distribution of P–CoO@PWC‐2 is obtained above 2 nm (Figure S3, Supporting Information). This change exhibits a significant increase in the mesoporosity of P–CoO@PWC‐2. The transmission electron microscopy (TEM) image of P–CoO@PWC‐2 (Figure 2d) confirms the encapsulation of Co species on the carbon plates. High‐resolution transmission electron microscopy (HRTEM) images (Figure 2e,f) reveal lattice strips with spacings of 0.215, 0.151, 0.245, and 0.340 nm, which match the *d*‐spacings for the (200), (220), and (111) planes of CoO and the (002) plane of C, respectively. The energy dispersive X‐ray spectroscopy (EDX) elemental mapping images reveal the uniform distribution of Co, C, O, and P over the whole electrode (Figure 2g,h). The crystal structures were ascertained by X‐ray diffraction (XRD) (Figure 2i; Figure S4, Supporting Information).^[^ ^31^, ^32^ ^]^ The obvious wide carbon peak at 2*ɵ* = 22.3–34.5 expresses that the carbon has somewhat low overall crystallinity. Usually this wide peak is ascribed to the long‐distance disorder of carbon nanocrystals with high crystalline degree. When Co species are doped on the carbon matrix, the added peaks for CoO@PWC at 36.5°, 42.4°, and 61.5° are attributed to the (111), (200), and (220) planes of CoO, respectively.^[^ ^33^, ^34^, ^35^ ^]^ After the surface‐phosphorization, the diffraction peaks well‐assigned to CoO in all the samples demonstrate that the incorporation of P does not alter the crystal structure of CoO particles. These results suggest that P atoms are only modified on the surface of CoO nanoparticles.

### 2.2 Structure and Chemical State Analysis

**Figure 3:**
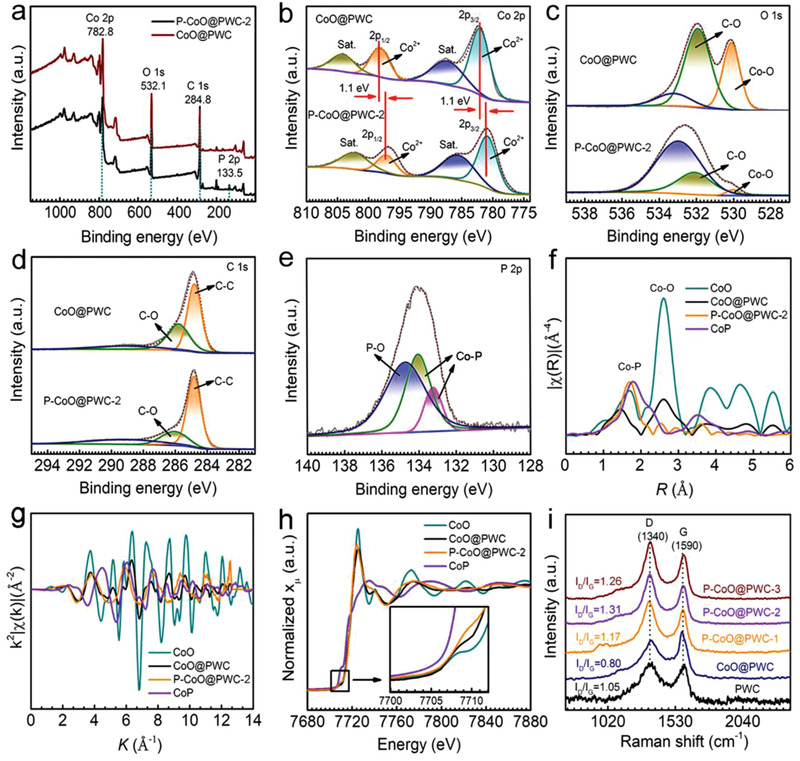
a) XPS survey spectra of CoO@PWC and P–CoO@PWC‐2. b–e) Co 2p, O 1s, C 1s, and P 2p spectra of CoO@PWC and P–CoO@PWC‐2. EXAFS spectra in the f) *R* and g) *k* spaces, and h) cobalt K‐edge XANES spectra of P–CoO@PWC‐2, CoO@PWC, CoO, and CoP. i) Raman spectra of P–CoO@PWC‐1, P–CoO@PWC‐2, P–CoO@PWC‐3, CoO@PWC, and PWC.

X‐ray photoelectron spectroscopy (XPS) shows that Co, O, C, and P are present in P–CoO@PWC‐2 (**Figure** **3**a). The appearance of the P peak in the P–CoO@PWC‐2 spectrum reveals that the surface of the sample is phosphorized successfully. The atomic content of each element is also clearly detected (Table S1, Supporting Information). The XPS spectra of Co 2p (Figure 3b) show that Co^2+^ exists in the catalysts.^[^ ^36^, ^37^ ^]^ It is noteworthy that the peak position of metallic Co 2p continually shifts to lower binding energy region with the incorporation of P atoms (Figure 3b). These shifts reflect that P atoms effectively modulate the electronic configuration of CoO@PWC. This modulation effect originates from the weaker electronegativity of P than O. The partial electrons transfer from P to Co and O results the lower Co valence in P–CoO@PWC‐2 than that of CoO@PWC.^[^ ^38^ ^]^ Focusing on the high‐resolution scanning of O 1s electrons for P–CoO@PWC‐2 (Figure 3c), the strength of the O–C and O–Co peaks is much weaker due to the increased concentration of defect sites after surface phosphorization, while the strength of the C═O peaks is stronger than that of CoO@PWC. As shown in Figure 3d, the peaks centered on 284.8 and 286.2 eV belong to C–C and C–O, respectively.^[^ ^39^, ^40^ ^]^ These signals originate in the biological carbon matrix. The fitted P 2p spectrum of P–CoO@PWC are divided into three peaks. The peak at 134.8 eV (Figure 3e) corresponds to the P—O bond.^[^ ^31^, ^32^, ^40^ ^]^ Additionally, the peaks at 133.0–134.3 eV confirm the formation of P—Co bonds in P–CoO@PWC‐2 via surface phosphorization.^[^ ^41^ ^]^ Notably, the absence of fitting peak at 132.6 eV proves that low‐temperature phosphorization leads no formation of P—C bonds.

Extended X‐ray absorption fine structure (EXAFS) spectroscopy and X‐ray absorption near‐edge structure (XANES) spectroscopy were used to elucidate the coordination environment and oxidation state of the Co species in P–CoO@PWC‐2. As shown in Figure 3f,g and Figure S5 in the Supporting Information, the EXAFS analysis spectra of CoO@PWC appear similar to that of CoO, while the peak amplitude and coordination number (Table S2, Supporting Information) of the first shell Co—O (1.3 Å) bond in P–CoO@PWC‐2 are lower than those of CoO@PWC.^[^ ^42^ ^]^ This peak type tending to be CoP reveals the decrease in the Co–O coordination number and the addition of Co–P coordination.^[^ ^43^, ^44^, ^45^ ^]^ The displacement of M—O and M—M bonds in P–CoO@PWC‐2 confirm the formation of an abundance of defects with the introduction of P. The structural parameters at the Co K‐edge were extracted by least‐squares EXAFS fitting (Figure S6, Supporting Information). Based on the coordination number (≈4) of M–O, typical Co–O_4_ and Co–O_3_P_1_ moieties predominate in CoO@PWC and P–CoO@PWC‐2, respectively. The special Co^2+^ coordination environment in P–CoO@PWC‐2 is reflected by a particular path located at 1.7–1.9 Å. The above result was further corroborated with the normalized XANES spectra at the Co K‐edge (Figure 3h). The similarity of the edge positions between CoO@PWC and P–CoO@PWC‐2 suggests an oxidized electronic structure for the Co species. Compared with CoO@PWC, the slight shifting of the Co K‐edge in P–CoO@PWC‐2 to a lower energy field suggests an average oxidation state between those in CoO and CoP. Co K‐edge wavelet transform (WT)‐EXAFS was utilized to investigate the atomic configuration of P–CoO@PWC‐2 (Figure S7, Supporting Information). Clearly, the CoO@PWC peaks correspond to CoO with only Co–O and Co–Co coordination, while P–CoO@PWC‐2 shows that the shell moves to the right and is between that of CoO and CoP. Through a comprehensive consideration of the Co–O_3_P_1_ contribution, the WT contour plots in P–CoO@PWC‐2 exhibit the maximum peak at 5.9 Å^−1^.^[^ ^46^ ^]^ The above results confirm the partial formation of new coordination modes in P–CoO@PWC‐2 via surface phosphorization.

Raman spectroscopy was applied to further investigate the physicochemical properties of the catalysts (PWC, CoO@PWC, P–CoO@PWC‐1, P–CoO@PWC‐2, and P–CoO@PWC‐3). The Raman spectra of the five samples display two distinct peaks (Figure 3i). The D bands represent defective/disordered carbon, and the G bands are ascribed to graphitic sp^2^ carbon. The *I*_D_/*I*_G_ can visually reflect the disorder parameter of carbon. PWC, CoO@PWC, P–CoO@PWC‐1, and P–CoO@PWC‐2 exhibit *I*_D_/*I*_G_ ratios of 1.05, 0.80, 1.17, and 1.31, respectively. The further increase in the *I*_D_/*I*_G_ ratio for P–CoO@PWC‐2 are due to the introduction of heteroatoms into the carbon network. Thus, more defects are synchronously caused by the CoO modification or surface phosphorization. The decrease in the *I*_D_/*I*_G_ ratio for P–CoO@PWC‐3 indicates that excessive phosphorization drives the carbon skeleton to reconfigure and result in promoted disordered structure.

### 2.3 Electrocatalytic Performances in ORR and OER

**Figure 4:**
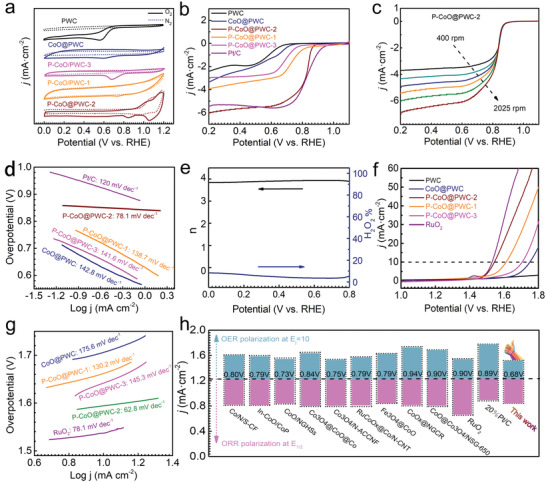
a) CV curves in N_2_‐ or O_2_‐saturated 0.1 m KOH electrolyte. b) LSV curves for the ORR at 1600 rpm. c) LSV curves at different rotating speeds. d) ORR Tafel plots of the catalysts. e) Calculated *n* and determined H_2_O_2_ yield (%) at various potentials based on the RRDE data of P–CoO@PWC‐2. f) OER polarization curves of the catalysts in O_2_‐saturated 0.1 m KOH. g) OER Tafel plots of the catalysts. h) Comparison of the *E*_gap_ values of our catalysts with that in the literature.

The bifunctional catalytic activities of P–CoO@PWC‐2 were then assessed in a three‐electrode system. The oxygen reduction peaks of these catalysts were observed in the cyclic voltammetry (CV) curves (**Figure** **4**a) that were obtained in a 0.1 m O_2_‐saturated KOH electrolyte. Notably, no such peaks appear in a 0.1 m N_2_‐saturated KOH electrolyte. The peak of P–CoO@PWC‐2 (0.84 V) is evidently higher than that of CoO@PWC (0.65 V), P–CoO@PWC‐1 (0.76 V), P–CoO@PWC‐3 (0.71 V), and PWC (0.60 V), indicating its significant catalytic activity for the ORR. The linear sweep voltammetry (LSV) curves in Figure 4b show P–CoO@PWC‐2 with a positive initial potential (*E*_0_, 0.91 V) and half‐wave potential (*E*_1/2_, 0.84 V), thereby showing remarkable ORR activity. In terms of E_1/2_, P–CoO@PWC‐2 surpasses most nonprecious metal electrocatalysts reported in the literature (Table S3, Supporting Information). Regarding the P–CoO@PWC family electrocatalysts, it is interesting to observe that the variation in the P content of the different P–CoO@PWC catalysts, which is realized by controlling the phosphorization duration, has a large effect on electrocatalytic performance. P–CoO@PWC‐2 is identified to be the best catalyst among the entire P–CoO@PWC family of catalysts. The LSV profiles at different rotational speeds were further evaluated in regard to the catalytic performance of the ORR (Figure 4c). P–CoO@PWC‐2 shows a well‐defined plateau of diffusion‐limited currents at all rotation speeds, where as expected, the current densities increase with an increasing rotation speed. This result is due to the expedited mass transfer of oxygen molecules from the electrolyte to the electrode surface.^[^ ^42^, ^47^ ^]^ Figure 4d shows that the Tafel slope for the ORR of P–CoO@PWC‐2 (78.1 mV dec^−1^) is much lower than that of Pt/C (120.0 mV dec^−1^), thereby showing high‐efficient ORR kinetics in molecular adsorption and activation processes. The electron transfer number of P–CoO@PWC‐2 was measured by a rotating ring–disk electrode (RRDE) (Figure S8, Supporting Information). From 0 to 0.8 V, the ring current is negligible. The number of electrons transferred (*n*) is between 3.80 and 3.95, corresponding to a yield of hydrogen peroxide (H_2_O_2_%) less than 10%. It is confirmed that O_2_ undergoes a typical four‐electron process on P–CoO@PWC‐2 during the ORR process (Figure 4e).

To evaluate the bifunctionality of P–CoO@PWC‐2, the OER performance was also analyzed through LSV profiles. In an alkaline electrolyte, to achieve a current density of 10 mA cm^−2^, P–CoO@PWC‐2 only requires an overpotential of 290 mV, which is smaller than that of CoO@PWC (525 mV), P–CoO@PWC‐1 (380 mV), and P–CoO@PWC‐3 (470 mV) (Figure 4f). The Tafel plot is usually employed to explore the OER kinetics, and a small Tafel slope implies favorable reaction kinetics in molecular adsorption and activation. P–CoO@PWC‐2 exhibits a Tafel slope value of 62.8 mV dec^−1^ (Figure 4g), which is remarkably lower than that of RuO_2_ (78.1 mV dec^−1^) and the remaining catalysts. This result indicates that the strong interaction of the surface phosphorus and CoO nanoparticles can effectively compensate for the electrical conductivity of P–CoO@PWC‐2. The facilitated charge transfer during ORR and OER, and regulated the adsorption/desorption process of oxygen‐related intermediates promote ORR and OER reaction kinetics. The potential difference Δ*E* between *E_j_*_/10_ and *E*_1/2_ comprehensively assesses the bifunctional activity of P–CoO@PWC‐2 (Figure 4h). Compared with Pt/C, RuO_2_ and the reported cobalt‐based catalysts (Table S3, Supporting Information), P–CoO@PWC‐2 possesses comparable or higher bifunctional activity. Considering that all the P–CoO@PWC catalysts share similar structural features, the excellent bifunctional activity of P–CoO@PWC‐2 can be reasonably ascribed to the incorporation of an optimal P amount. This finding might be attributed to the fact that an insufficient phosphorization extent (15 min) endows the as‐converted P–CoO@PWC‐1 catalyst with a low P content, thus increasing the charge exchange resistance. In contrast, excessive phosphorization (60 min) may lead to a high P content and fewer catalytically active sites as well as high electrical resistance in the resultant P–CoO@PWC‐3 catalyst. Accordingly, P–CoO@PWC‐2, which is obtained at a moderate phosphorization time (30 min), demonstrates an abundance of active sites and an optimal electronic–electrical structure, thereby showing the highest bifunctional activity (Figure S9, Supporting Information).

To evaluate the application prospects of P–CoO@PWC‐2 in ZABs, its methanol resistance and long‐term stability were studied by chronoamperometry (*i*–*t*) measurements. After the addition of methanol to the electrolyte, the ORR current of Pt/C decreases rapidly and remains at only 53% after 1800 s, while the ORR current of the P–CoO@PWC‐2 catalyst maintains 91% of its initial value (Figure S10a, Supporting Information). The occupation of catalytic active sites by methanol molecules produces an oxidation current and inhibits the ORR process on 20% Pt/C. While P–CoO@PWC‐2 has a higher methanol resistance than 20% Pt/C. After running for 12 h, P–CoO@PWC‐2 shows better electrocatalytic stabilities during both the OER and ORR than those of 20% Pt/C and RuO_2_ catalysts (Figure S10, Supporting Information). Favorable chemical stability is beneficial to the electrochemical stability of catalysts.^[^ ^48^ ^]^ The accelerated degradation test (ADT) was then implemented by continuous CV tests in 0.1 m O_2_‐saturated KOH electrolyte for 10 000 cycles. After the ADT test, the robust stability of P–CoO@PWC‐2 is confirmed by the slight shifting (almost 8 mV) of *E*_1/2_ (Figure S11, Supporting Information). The results show that the stability of the prepared P–CoO@PWC‐2 is much higher than that of 20% Pt/C. The stability of the electrochemical properties of P–CoO@PWC‐2 is beneficial to its practical application.

After the durability tests, the morphology and surface properties of P–CoO@PWC‐2 were further investigated to prove the structural and chemical stability of P–CoO@PWC‐2. The SEM and TEM images of the recovered catalyst reveal that the architecture is well preserved, and most of the cubic nanoparticles are still embedded in P–CoO@PWC‐2 (Figure S12a–c, Supporting Information). The HRTEM (Figure S12d, Supporting Information) image shows the same situation with spacings of 0.215, 0.151, 0.245, and 0.340 nm, matching the *d*‐spacings of the (200), (220), and (111) planes of CoO and the (002) plane of C, respectively. XPS spectra were also acquired and are shown in Figures S13 and S14 in the Supporting Information. The XPS spectra reveal that Co 2p, O 1s, C 1s, and P 2p still exist in the previous valence state and shift to higher binding energies after the stability tests. This result shows that P–CoO and PWC are involved in the electrochemical reactions. The co‐involved P–CoO and PWC is beneficial for the synergistic effect and is responsible for the excellent bifunctional performance of P–CoO@PWC‐2.

The prominent electrocatalytic activity and durability of P–CoO@PWC‐2 are ascribed to several factors: 1) the surface phosphorus modulation on the surface polarities and electronic properties, 2) the high specific and electroactive surface areas of P–CoO@PWC‐2 due to its porous structure, 3) the strong interaction between P–CoO nanoparticles and PWC, and 4) the low O_2_ adsorption and dissociation energy resulting from the surface phosphorus induction.

### 2.4 Theoretical Insights into Catalytic Mechanism

**Figure 5:**
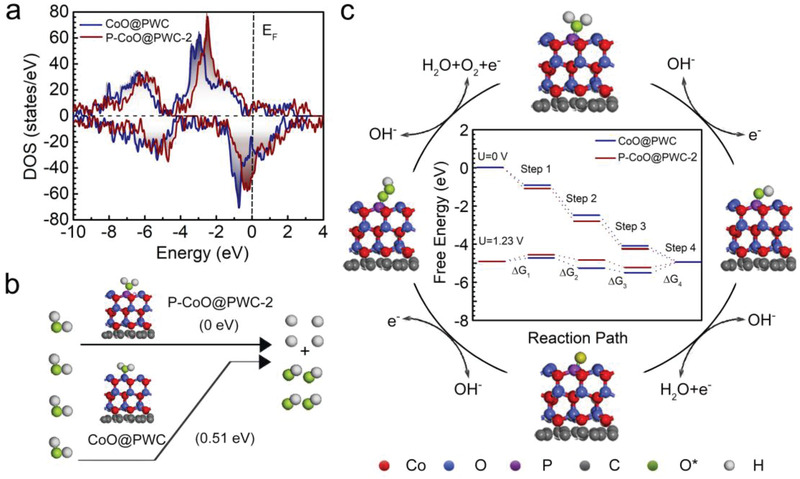
a) Density of states of CoO@PWC and P–CoO@PWC‐2. b) Adsorption configurations of water on the CoO@PWC and P–CoO@PWC‐2 surfaces and schematic energy diagrams for water dissociation. c) Free energy diagrams for the ORR and OER at different electrode potentials on CoO@PWC and P–CoO@PWC‐2 through an oxygen associative mechanism. The reaction pathways for the ORR listed in this panel are Step 1: O_2_ (g) + * + H_2_O (l) + e^−^ ↔ OOH* + OH^−^, Step 2: OOH* + e^−^ ↔ O* + OH^−^, Step 3: O* + H_2_O (l) + e^−^ ↔ OH* + OH^−^, and Step 4: OH* + e^−^ ↔ * + OH^−^; the OER occurs in reverse from Step 4 to Step 1.

To test the above conjecture, density functional theory (DFT) calculations were used to survey the free energy of the four‐electron ORR and OER mechanism. After the introduction of P atoms, the electronic state of P–CoO@PWC close to the Fermi level clearly increases, revealing a high carrier density and favorable charge transfer in the electrocatalytic process of P–CoO@PWC‐2 (**Figure** **5**a). As illustrated in Figure 5b, water molecules are chemically adsorbed and can be dissociated into H* and OH* with an energy barrier of 0.51 eV on CoO@PWC. Surprisingly, water molecules demonstrate dissociative adsorption on P–CoO@PWC‐2, in which they spontaneously dissociate and form H* and OH*, implying a superior property of water dissociation for proton feeding in comparison with that on CoO@PWC. The advantage of P–CoO@PWC‐2 in regard to water activation is the key to improving the ORR performance. The O_2_ molecules adsorbed on the surface of the ORR electrocatalysts decompose into two O atoms by the oxygen dissociation mechanism or combine with water molecules to form OOH* intermediates by the association mechanism.^[^ ^40^, ^41^ ^]^ Figure 5c shows the free energies of various oxygen‐containing intermediates for the ORR and OER on CoO@PWC and P–CoO@PWC‐2. The results indicate that the elementary reactions of the ORR/OER are exothermic/endothermic, respectively, when the electrode potential is 0 V (corresponding to the short‐circuit state of the battery), demonstrating the feasibility of both the OER and ORR reactions with P–CoO@PWC‐2 as a bifunctional electrocatalyst.^[^ ^49^ ^]^ The initial O_2_ adsorption and final OH* desorption steps are clearly endothermic, but the intermediate two steps are still close to being downhill when the electrode potential increases to 1.23 V. The rate‐limiting step of the ORR is confirmed by the smallest Gibbs free energy change Δ*G*_2_ of the four reaction steps (the separation of OOH*).^[^ ^47^, ^48^, ^49^ ^]^ Furthermore, a similar analysis shows that the potential‐determining step of the OER on CoO@PWC is Step 1 for O_2_ generation, exhibiting a higher external force to drive the whole process than that on P–CoO@PWC‐2. When P atoms are introduced into CoO@PWC, the free energy of oxygen adsorption increases significantly. P–CoO@PWC‐2 shows a low energy gap. Thus, the incorporation of P atoms is advantageous to overcome the slow OH* desorption step because P atoms provide electrons to CoO, thus weakening the binding force between adsorbed OH*. Therefore, the DFT calculations correlated with the experimental results verify the synergistic coupling function between the P atoms and CoO nanoparticles. These facts result in a positive surface phosphorus‐induced electronic environment and an improvement in the catalytic performances during the ORR and OER.

### 2.5 Applications in ZABs

**Figure 6:**
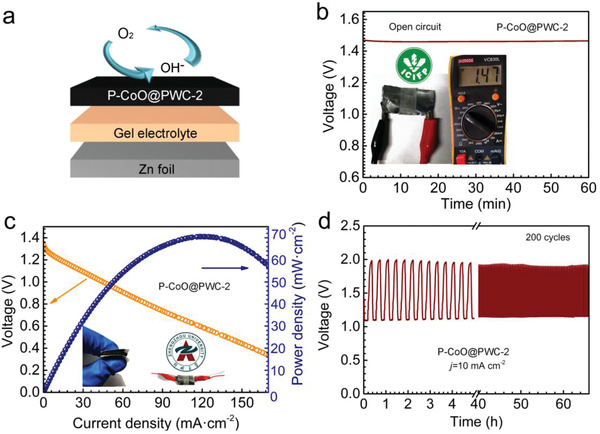
Performance of the quasi‐solid‐state RZABs with P–CoO@PWC‐2 as the monolithic air electrode. a) Schematic diagram of a quasi‐solid‐state RZAB. b) Open‐circuit plots of a single battery; the inset shows the digital image. c) Discharge curve and power density plot. d) Cyclic galvanostatic charge–discharge plots at 10 mA cm^−2^.

With the growing need for flexible energy storage facilities, quasi‐solid‐state ZABs are becoming a prospective alternative.^[^ ^21^, ^50^ ^]^ P–CoO@PWC‐2 shows high promise for application in quasi‐solid‐state RZABs (**Figure** **6**a). Quasi‐solid‐state ZABs were packaged with a monolithic P–CoO@PWC‐2 cathode, polyvinyl alcohol (PVA) gel electrolyte, Zn foil anode, and nickel foam current collector (Figure S15, Supporting Information). The ZAB exhibits a high open‐circuit voltage of 1.47 V (Figure 6b), along with a peak power density of 73 mW cm^−2^ (Figure 6c). Figure 6d displays stable charge (1.97 V) and discharge (1.14 V) voltages at a current density of 10 mA cm^−2^ and a slight voltage change after 200 cycles, which lasted for 67 h. The superior battery performances of P–CoO@PWC‐2 are attributed to its excellent catalytic activity toward the ORR/OER and its three‐phase interface. The abundance of transport channels promote the diffusion of reactive species.

**Figure 7:**
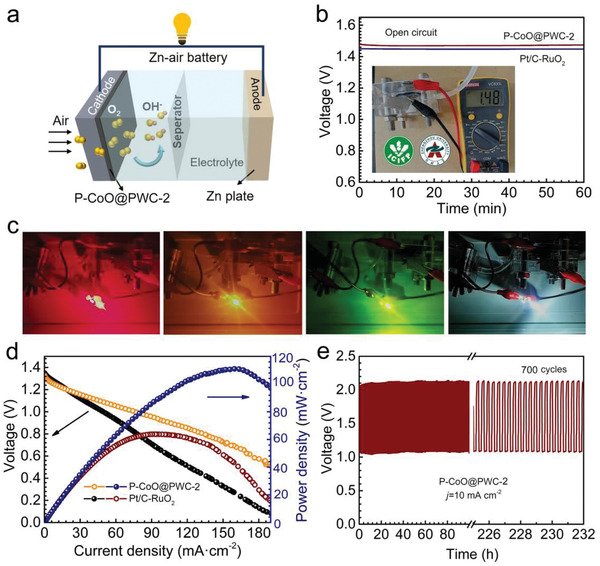
a) Simple illustration of aqueous ZAB using P–CoO@PWC‐2 as an air electrode. b) Open‐circuit plots of a single battery; the inset shows the digital image. c) Red, white, yellow, and green LEDs powered by two‐series batteries based on P–CoO@PWC‐2. d) Polarization curves and power density of ZABs based on P–CoO@PWC‐2 and Pt/C–RuO_2_. e) Long‐term cycling stability of RZABs based on P–CoO@PWC‐2.

P–CoO@PWC‐2 was also used to fabricate air electrodes for aqueous ZABs (**Figure** **7**a). Pt/C–RuO_2_ was applied as an air electrode to manufacture ZABs for comparison. The ZAB based on P–CoO@PWC‐2 exhibits a high open‐circuit voltage of 1.48 V, corresponding to its excellent ORR performance (Figure 7b). Moreover, the two RZABs based on P–CoO@PWC‐2 were used to power a light‐emitting diode (LED) (Figure 7c). Figure 7d shows the discharge polarization curves and the power density of ZABs. The ZAB based on P–CoO@PWC‐2 exhibits a maximum power density of 113 mW cm^−2^ and a high current density of 178 mA cm^−2^, which is better than the battery using Pt/C–RuO_2_. To further assess the cycling stability of P–CoO@PWC‐2, the ZABs based on P–CoO@PWC‐2 and Pt/C–RuO_2_ were cycled at 10 mA cm^−2^ (Figure 7e). The RZABs based on P–CoO@PWC‐2 maintain a nearly unchanged charge–discharge voltage gap (0.87 V at the 1st h and 0.91 V at the 232nd h) after 700 cycles. This voltage gap higher than that of Pt/C–RuO_2_ (1.11 V at the 1st h and 1.62 V at the 91st h; Figure S16, Supporting Information) confirms the outstanding electrocatalytic performances of P–CoO@PWC‐2. Additionally, the performance of P–CoO@PWC‐2 exceeds most reported nonprecious catalysts (Table S3, Supporting Information), thereby revealing the superb application potential of catalytically active carbon in ZABs.

## 3 Conclusions

In conclusion, surface phosphorus‐induced CoO nanoparticles coupled with carbon plates (P–CoO@PWC‐2) were proposed as monolithic electrodes to demonstrate highly efficient bifunctional oxygen electrocatalysis for superior performance in ZABs. The surface phosphorus‐induced catalytic activity and stability toward the ORR and OER were comparable to those of Pt/C and RuO_2_. Experimental research and DFT calculations revealed that the incorporation of P atoms onto the surface of P–CoO@PWC‐2 adjusted its electronic structure to facilitate the dissociation of water molecules and the activation of oxygen molecules, thus promoting its bifunctional catalytic activity. The quasi‐solid‐state ZABs using a monolithic P–CoO@PWC‐2 electrode displayed a high open‐circuit potential of 1.48 V, a maximum power density of 73 mW cm^−2^, and excellent charge–discharge stability. This work manifests a surface phosphorization‐monolithic strategy for the fabrication of carbon material electrodes. This strategy can be further extended to other monolithic catalytically active carbon composites for storage systems and various energy conversions, such as other metal–air batteries, ion batteries, and supercapacitors.

## 4 Experimental Section

### Materials

Paulownia wood was obtained from Henan Province, China. The wood samples were cut into the same size, 30 mm × 30 mm × 3 mm (radial × tangential × longitudinal). Cobalt(II) chloride hexahydrate (CoCl_2_·6H_2_O, ≥99%), hexamethylenetetramine (HMT, ≥99%), sodium hypophosphite (NaH_2_PO_2_, 99%), ruthenium oxide powder (RuO_2_, 99%), and platinum on carbon (Pt/C, 20 wt% loading) were purchased from Sigma‐Aldrich. Deionized water (DI water, 18.2 MΩ cm) was used for all aqueous solutions. All chemical reagents were of analytical grade, and used without further purification.

### Preparation of PWC

Typically, dried paulownia wood chips were calcined in 800 °C under Ar atmosphere for 2 h, and the carbon plates were marked as PWC.

### Preparation of CoO@PWC

Co(OH)_2_@PWC intermediate was synthesized on carbon plate. In a typical synthesis of the composites, CoCl_2_·6H_2_O (0.143 g), HMT (1.0 g), and DI water (20 mL) were mixed to obtain a pink solution. PWC was completely immersed into the above solution, heated for 12 h at 90 °C, dried at 60 °C for complete dehydration, and it was named as Co(OH)_2_@PWC. The CoO@PWC was synthesized at 350 °C for 1 h by placing Co(OH)_2_@PWC in a tube furnace under N_2_ atmosphere.

### Preparation of P–CoO@PWC

CoO@PWC and NaH_2_PO_2_ (1:20) were placed at two porcelain boats, and NaH_2_PO_2_ was placed upstream of tube furnace. Then, these porcelain boats were heated to 350 °C in Ar atmosphere. The samples heated for 15, 30, and 60 min were expressed as P–CoO@PWC‐1, P–CoO@PWC‐2, and P–CoO@PWC‐3, respectively. Each sample was prepared and tested at least three times.

### Material Characterizations

The morphologies of the prepared materials were tested by TEM (FEI Tecnai G^2^ F20 S‐TWIN electron microscope, operating at 200 kV) and field emission SEM (ZEISS Sigma 500). EDX was used to get information about element distribution. From the adsorption branch of isotherm curves in the *P*/*P*° range between 0.05 and 0.35, the specific surface areas (*S*_BET_) of BMNC were calculated by the multipoint Brunauer‐Emmett‐Teller (BET) method. The pore size distribution was evaluated by the nonlocalized density function theory (NLDFT). The N_2_ sorption isotherms were measured on surface area and pore size analyzer (ASAP2420‐4MP, Micromeritics, USA) at 77 K. The phase structures of products were characterized by XRD (Bruker D8 Advance with Cu K*α*, *λ* = 1.5418 Å). Co K‐edge X‐ray absorption spectroscopy (XAS) was acquired under ambient conditions in transition mode at beamline 1W1B of Beijing Synchrotron Radiation Facility (BSRF), using a Si (111) double‐crystal monochromator. The storage ring of BSRF was operated at 1.5 GeV with a maximum current of 250 mA in decay mode. The DEMETER software package (ATHENA and ARTEMIS) was used for X‐ray absorption fine structure (XAFS) data analysis of CoO@PWC and P–CoO@PWC‐2 absorption spectra in comparison with standards and relative to Co foil, CoO, and CoP. XPS was recorded on a PHI quantera SXM spectrometer with an Al K*α* = 1486.6 eV excitation source, where binding energies were calibrated by referencing the C 1s peak (284.8 eV) to reduce the sample charge effect. Raman spectroscopy was obtained by using an HR Evolution Raman Spectrometer (Horiba Scientific, France) with excitation from the 514 nm line of an Ar‐ion laser with a power of about 5 mW. The present first‐principle DFT calculations were performed with the projector augmented wave (PAW) method. The exchange‐functional was treated using the generalized gradient approximation (GGA) of Perdew–Burke–Ernzerhof (PBE) functional. The cut‐off energy of the plane‐wave basis was set at 450 eV to optimize calculations of atoms and cell optimization. The vacuum spacing in a direction perpendicular to the plane of the catalyst was at least 10 Å. The Brillouin zone integration was performed using 3 × 3 × 1 Monkhorst–Pack *k*‐point sampling for a primitive cell. The self‐consistent calculations applied a convergence energy threshold of 10^−3^ eV. The equilibrium lattice constants were optimized with maximum stress on each atom within 0.02 eV Å^−1^. The Hubbard U (DFT+U) corrections for 3d transition metal was set according to the literature. The free energy was calculated using the equation: *G* = *E* + ZPE − TS, where *G*, *E*, ZPE, and TS are the free energy, total energy from DFT calculations, zero point energy, and entropic contributions (*T* was set to be 300 K), respectively.

### Electrochemical Tests

All electrochemical measurements were performed on a CHI760E electrochemical workstation in a three‐electrode system, taking Pt wire as the counter electrode and Ag/AgCl electrode as the reference. The working electrode was prepared as follows: the dispersion consisting of catalysts (3 mg), ethanol (500 µL), and Nafion (50 µL) was sonicated for 60 min. Then, as‐prepared homogeneous dispersion (15 µL) was dropped onto glassy carbon of rotating disk electrode (RDE) or RRDE. After drying at room temperature, the glassy carbon of RDE or RRDE coated with catalysts was applied as the working electrode. All potential values were converted to potential versus reversible hydrogen electrode (RHE) according to the following calculation

(1) $$E_{\mathrm{versus}\;\mathrm{RHE}}=E_{\mathrm{versus}\;\mathrm{Ag}/\mathrm{Agcl}}+{E^{\theta }}_{\mathrm{versus}\;\mathrm{Ag}/\mathrm{Agcl}}+0.059\;\mathrm{pH}$$

Before the electrochemical test, the glass reaction vessel and the vent should be boiled with deionized water. After drying, the glass cell was immersed in strong alkaline solution to remove any possible metallic impurities. CV was performed in the voltage range of 0–1.2 V (vs RHE) for all the samples. The scanning speed was set as 50 mV s^−1^. The CV test was carried out under nitrogen and oxygen flow, respectively. LSV test was performed in O_2_‐saturated 0.1 m KOH solution at different rotating speeds. The test range was 0.2–1.2 V (vs RHE) and the scan rate was 5 mV s^−1^. The velocity of airflow was adjusted to 40 sccm. ADT was tested in O_2_‐saturated 0.1 m KOH solution. LSV of the system after 10 000 CV cycles (from 1.2 to 0 V vs RHE, at the scan rate of 50 mV s^−1^) showed the shift of the half‐wave potential. The *i*–*t* test was performed at 0.8 V for 22 h in O_2_‐saturated 0.1 m KOH solution at 1600 rpm. The *i*–*t* responses were recorded with or without methanol at 1600 rpm, and the concentration of methanol was about 1 m. To get the current density, the as‐obtained faradaic current was normalized using the geometric surface area. The *I*_D_ and *I*_R_ were tested with the RRDE in 0.1 m KOH at 1600 rpm and the potential of the ring was kept at 1.27 V (vs RHE). The electron‐transfer number (*n*) and the yield of hydrogen peroxide released during ORR were calculated based on the following equations

(2) $$n=4\times \frac{I_{D}}{I_{D}+I_{R}/N}$$

(3) $$\left[H_{2}O_{2}\right]\%=200\times \frac{I_{R}/N}{I_{R}/N+I_{D}}$$

where *I*_D_ is the disk current, *I*_R_ is the ring current, and *N* is the collection coefficient of the Pt ring (*N* = 0.37). Furthermore, commercial Pt/C electrocatalysts were used as a reference to evaluate the electrocatalytic performance of various samples.

The LSV test for OER was also performed in N_2_‐saturated KOH solution. A Pt wire was used as the counter electrode and an Ag/AgCl electrode was used as the reference. The working electrode was fabricated via a similar method to ORR test. The measurement was performed in 1.0–1.8 V (vs RHE) with the scan rate of 5 mV s^−1^. The OER stability was determined by *i*–*t* responses at 1600 rpm at the potential of 1.56 V.

The aqueous RZABs were assembled with catalyst as the air electrode, a polished Zn plate as the anode, and KOH (6.0 m) containing an aqueous Zn acetate (0.2 m) solution as the electrolyte. The quasi‐solid‐state ZABs were fabricated with a polished Zn foil as anode, P–CoO@PWC‐2 as freestanding air cathode, and PVA as electrolyte. The PVA powder (1799) gel electrolyte was prepared via heating, freezing, and thawing procedure. The discharge/charge cycling of RZABs was conducted using an automatic battery testing system (Neware CT‐3008) with 20 min for each cycle (discharge, 10 min. charge, 10 min) at 10 mA. Polarization data (*v*–*i*) were collected using LSV at a scan rate of 10 mV s^−1^. The current and power density curves were calculated from the LSV curves. All tests were performed in a natural environment at room temperature.

## Conflict of Interest

The authors declare no conflict of interest.

## Supporting information

Supporting Information

## Data Availability

Research data are not shared.
