# Supplementary material for: Surface Phosphorus‐Induced CoO Coupling to Monolithic Carbon for Efficient Air Electrode of Quasi‐Solid‐State Zn–Air Batteries
Source: Adv Sci (Weinh). 2021 Aug 8;8(19):2101314. doi: 10.1002/advs.202101314 (PMC8498900; doi:10.1002/advs.202101314)
Supplement: Supplementary file 1 — Supporting Information [file ADVS-8-2101314-s001.pdf]

© 2021 Wiley-VCH GmbH

ADVANCED SCIENCE
Open Access

Supporting Information

for *Adv. Sci.,* DOI: 10.1002/advs.202101314

Surface Phosphorus–Induced CoO Coupling to Monolithic Carbon for Efficient Air Electrode of Quasi-Solid-State Zn–Air Batteries

Huan Liu, Yanyan Liu, Sehrish Mehdi, Xianli Wu, Tao Liu, Benji Zhou, Pengxiang Zhang, Jianchun Jiang* and Baojun Li*

Supporting Information

Surface Phosphorus–Induced CoO Coupling to Monolithic Carbon for Efficient Air Electrode of Quasi-Solid-State Zn–Air Batteries

Huan Liu, Yanyan Liu, Sehrish Mehdi, Xianli Wu, Tao Liu, Benji Zhou, Pengxiang Zhang, Jianchun Jiang* and Baojun Li*

Miss H. Liu, Prof. Y. Liu, Miss S. Mehdi, Prof. X. Wu, Mr. B. Zhou, Mr. P. Zhang, Prof. B. Li
College of Chemistry, Zhengzhou University, 100 Science Road, Zhengzhou 450001, P. R. China
Miss H. Liu, Prof. Y. Liu, Prof. J. Jiang
Institute of Chemical Industry of Forest Products, CAF, National Engineering Lab for Biomass Chemical Utilization, Key and Open Lab on Forest Chemical Engineering, SFA, 16 Suojinwucun, Nanjing 210042, P. R. China
* Corresponding Author. E–mail: lyylhs180208@163.com; jiangjc@icifp.cn
Prof. Y. Liu
College of Science, Henan Agricultural University, Zhengzhou, Henan 450002, P.R. China
Mr. T. Liu
CAS Key Laboratory for Biomedical Effects of Nanomaterials and Nanosafety, National Center for Nanoscience and Technology, Beijing 100190, P. R. China

Total number of pages: 14

Total number of figures: 16

Total number of tables: 3

Table of contents

Figure S1 .... S3
Figure S2 .... S3
Figure S3 .... S4
Figure S4 .... S4
Table S1........ S5
Figure S5 .... S5
Figure S6 .... S6
Figure S7 .... S7
Table S2........ S8
Figure S8 .... S8
Table S3........ S9
Figure S9 .... S10
Figure S10 .... S10
Figure S11 .... S11
Figure S12 .... S11
Figure S13 .... S12
Figure S14 .... S12
Figure S15 .... S13
Figure S16 .... S14
Reference........ S14

Supplementary Figures

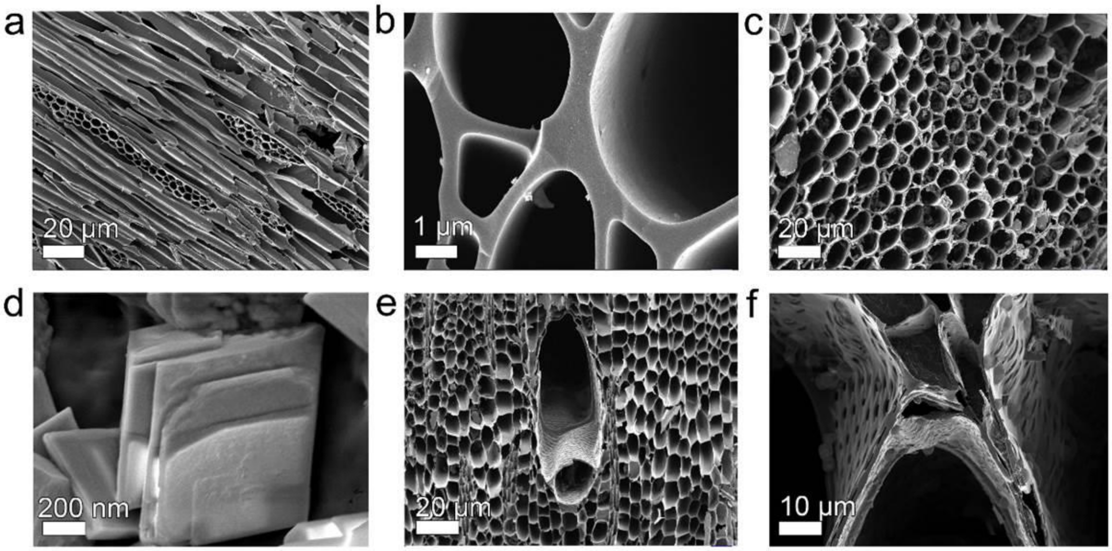

Figure S1. SEM images of (a–b) PWC, (c) CoO@PWC, (d) $Co(OH)_2$@PWC and (e–f) P–CoO@PWC–2.

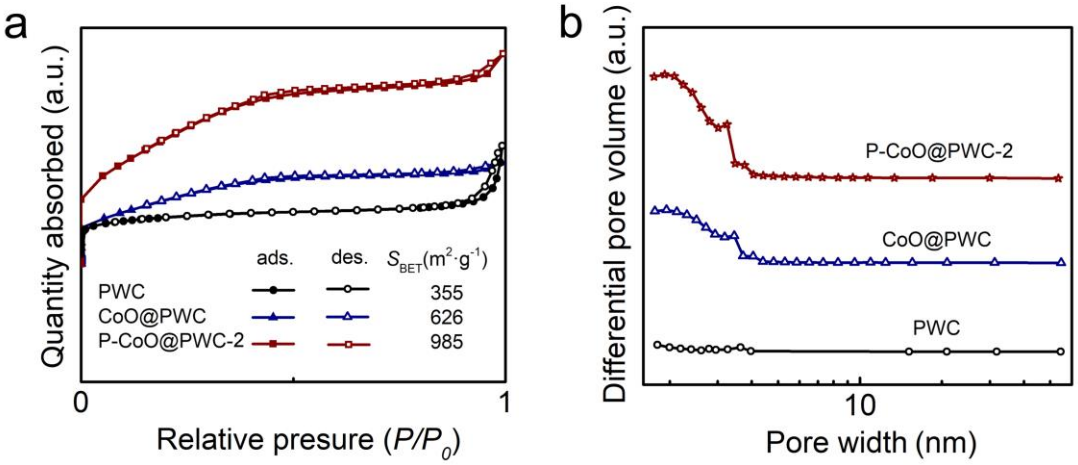

Figure S2. (a) Nitrogen adsorption–desorption isotherms and (b) pore size distribution of PWC, CoO@PWC, P–CoO@PWC–2.

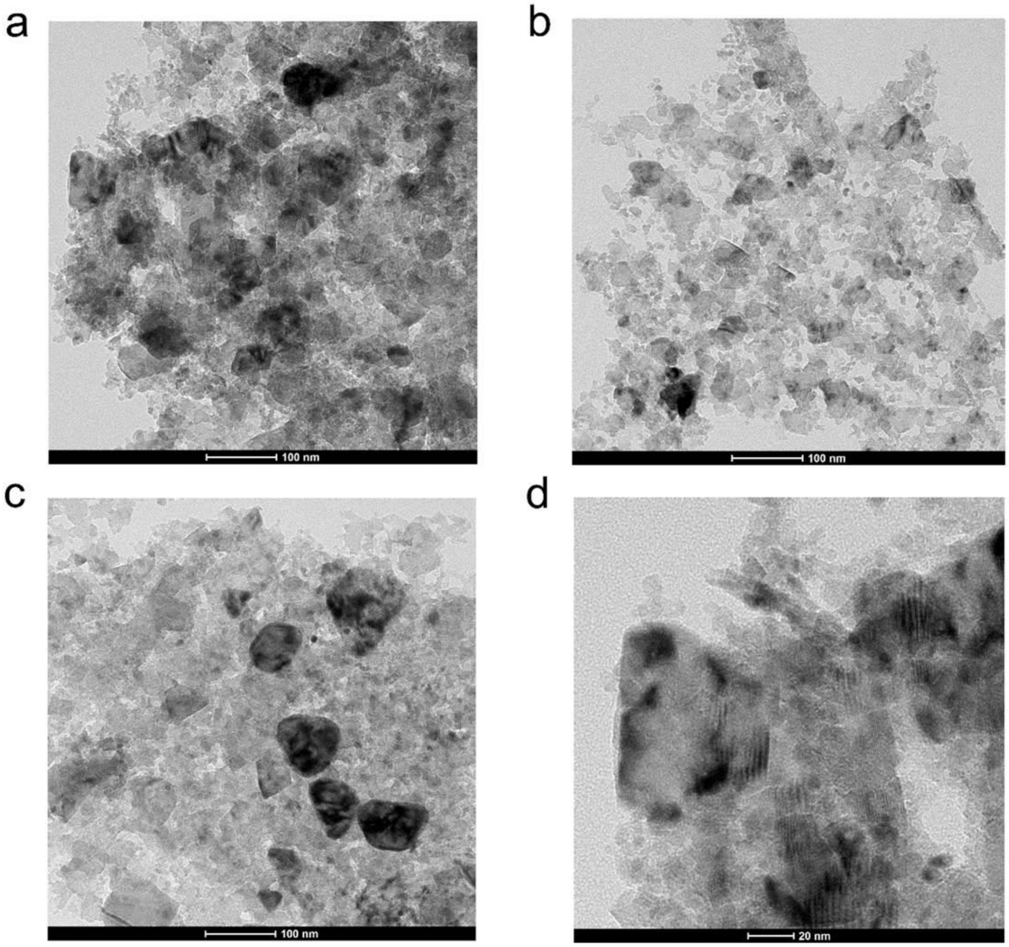

Figure S3. (a–c) TEM and (d) HRTEM images of CoO@PWC.

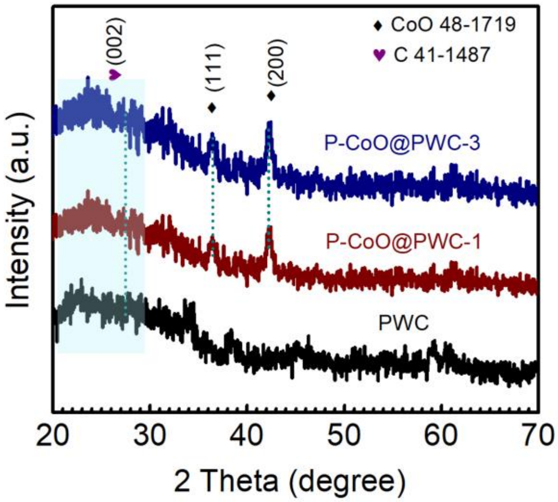

Figure S4. XRD patterns of PWC, P–CoO@PWC–1 and P–CoO@PWC–3.

Table S1. The atomic percentage of each element in various carbon electrodes obtained by XPS.

Electrode	C (at%)	Co (at%)	P (at%)	O (at%)
**PWC**	89.58	–	–	–
**CoO@PWC**	81.70	8.89	–	9.07
**P–CoO@PWC–1**	82.85	8.41	1.07	7.56
**P–CoO@PWC–2**	82.30	8.38	2.26	6.89
**P–CoO@PWC–3**	81.63	8.34	4.58	5.27

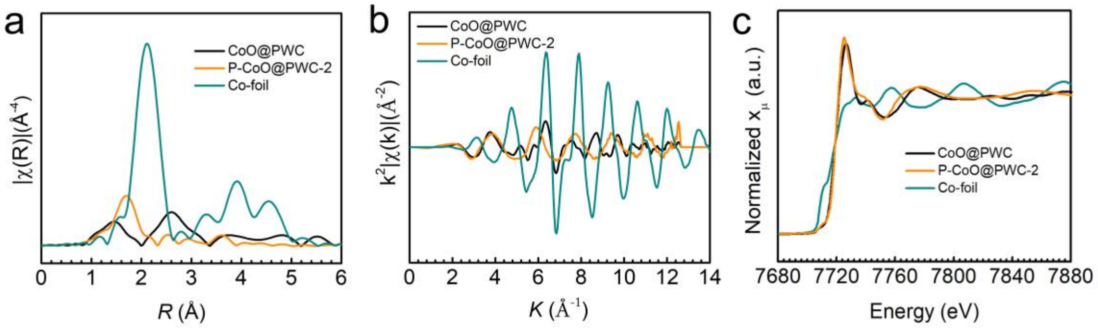

Figure S5. EXAFS spectra in R (a) and k (b) spaces and (c) Cobalt K edge XANES spectra of P–CoO@PWC–2, CoO@PWC and Co–foil, respectively.

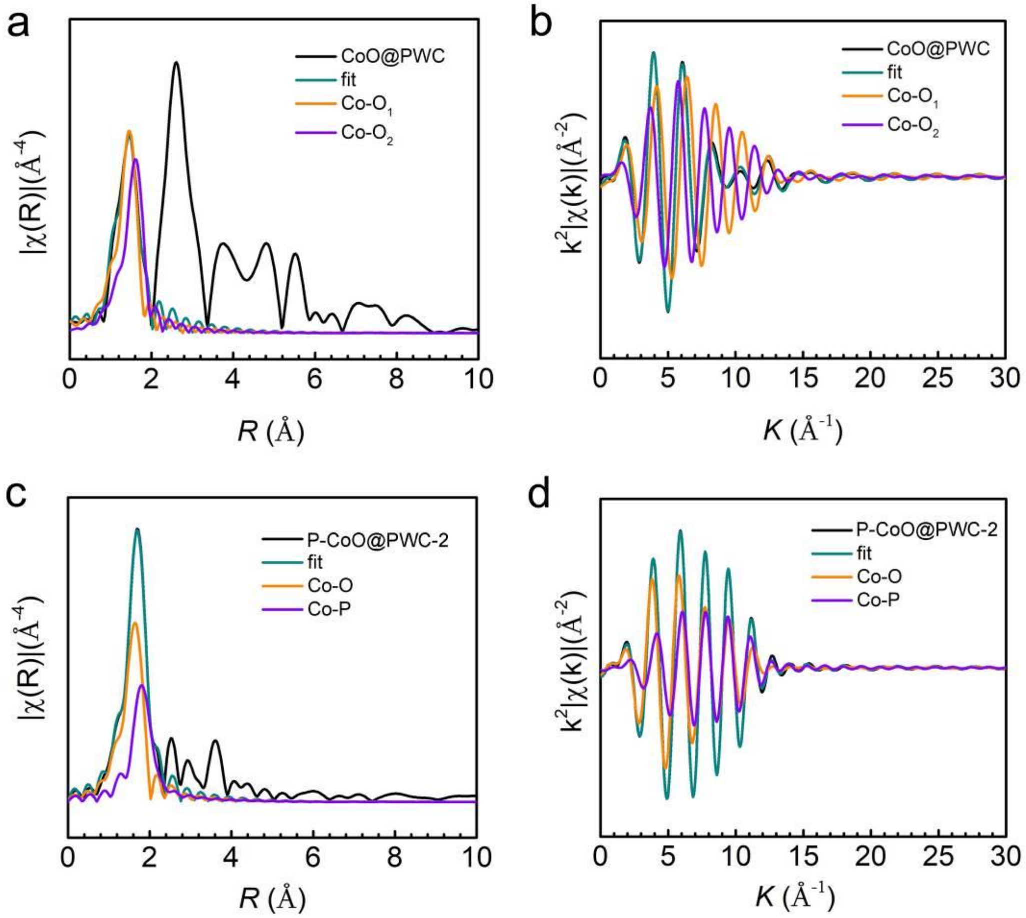

Figure S6. EXAFS fitting results for (a-b) CoO@PWC and (c-d) P–CoO@PWC–2 samples.

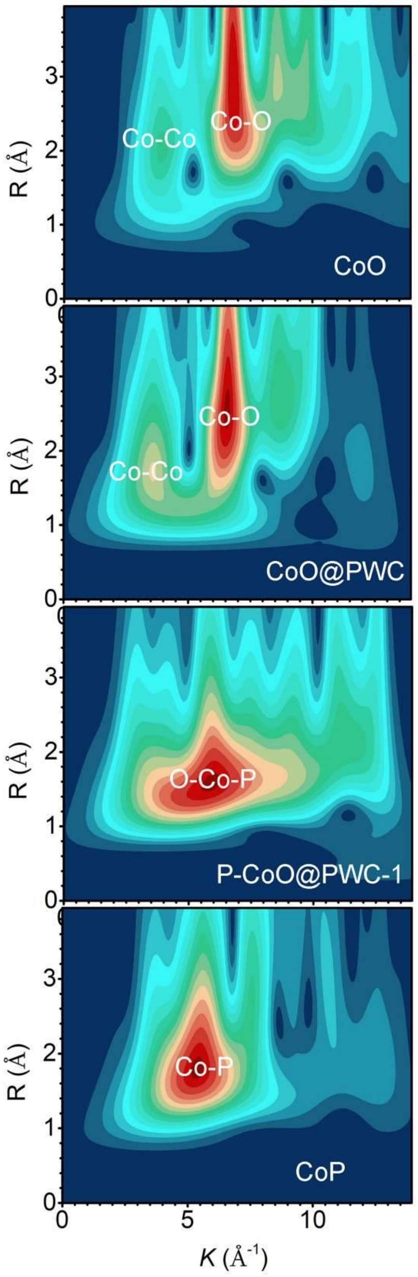

Figure S7. WT–EXAFS plots of CoO, CoO@PWC, P–CoO@PWC–2 and CoP, respectively.

Table S2. EXAFS fitting results for CoO@PWC and P–CoO@PWC–2.

Sample	Path	CN	R (Å)	σ^2(10–3Å^2)	ΔE_0 (eV)	R–factor
CoO@PWC	Co–O_1	2.1±0.2	1.86±0.01	4.8±0.9	–8.4±0.8	0.012
	Co–O_2	2.2±0.5	1.94±0.02			
P–CoO@PWC–2	Co–O	3.1±0.1	2.10±0.01	5.9±0.3	–2.0±0.4	0.001
	Co–P	1.0±0.1	2.27±0.02	2.7±0.3	–9.3±0.7	

CN: coordination number, R: the distance between the absorber and scatterer atoms, σ^2: Debye–Waller factor to account for thermal and structural disorders, ΔE_0: inner potential correction.

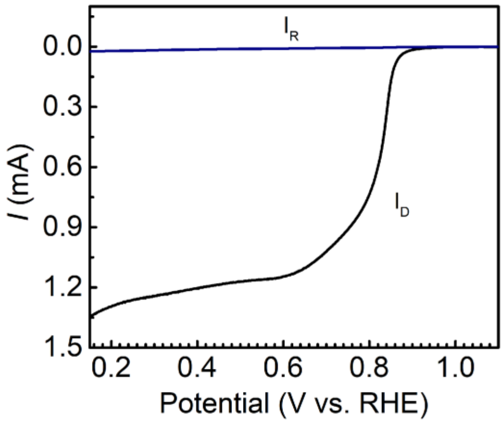

Figure S8. The disk and ring currents obtained with LSV on a RRDE for P–CoO@PWC–2.

Table S3. Summary of the catalytic activities of the reported composite electrocatalysts in 0.1 M KOH.

Catalyst	$E_{ORRonset}$ [V]	$E_{ORR1/2}$ [V]	E_{OER}[V] (j=10 mA·cm^{-2})	△E ($E_{j=10}$–$E_{1/2}$) [V]	RZABs Cycles/time	Ref.
P–CoO@PWC–2	0.91	0.84	1.52	0.68	700/232h	This work
$RuCoO_x$@Co/N–CNT	0.85	0.79	1.58	0.79	200/34h	[1]
Co_2P–Co_3O_4/rGO/C	0.91	0.80	–	–	–	[2]
In–CoO/CoP FNS	0.94	0.81	1.60	0.79	205/136h	[3]
CoO/NGHSs	0.92	0.83	1.56	0.73	–	[4]
VS–Co–2%	0.87	0.77	–	–	–	[5]
Co_3O_4@CoO@Co	0.91	0.79	1.65	0.84	–	[6]
Co_3O_4/N–ACCNF	0.98	0.79	1.54	0.75	240/80h	[7]
Co/N/S–CF	0.92	0.81	1.61	0.80	–	[8]
Fe_3O_4@CoO NCs	0.953	0.839	1.633	0.794	200/–	[9]
CoO_x@NGCR	0.91	0.80	1.74	0.94	–/17h	[10]
CoO@Co_3O_4/NSG–650	0.91	0.79	1.69	0.90	–	[11]

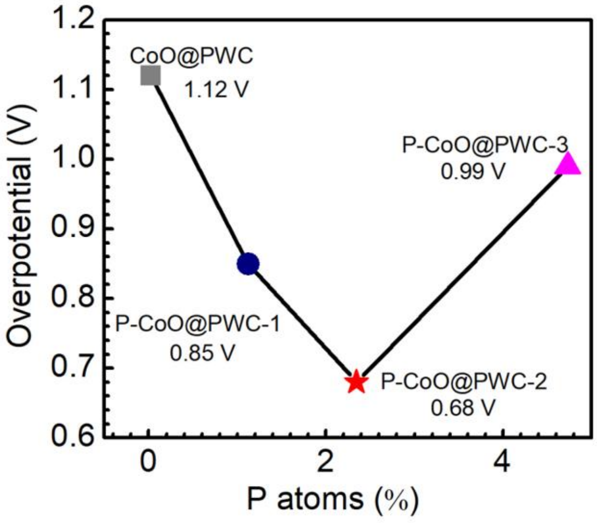

Figure S9. The relationship between atomic content of catalysts and performance.

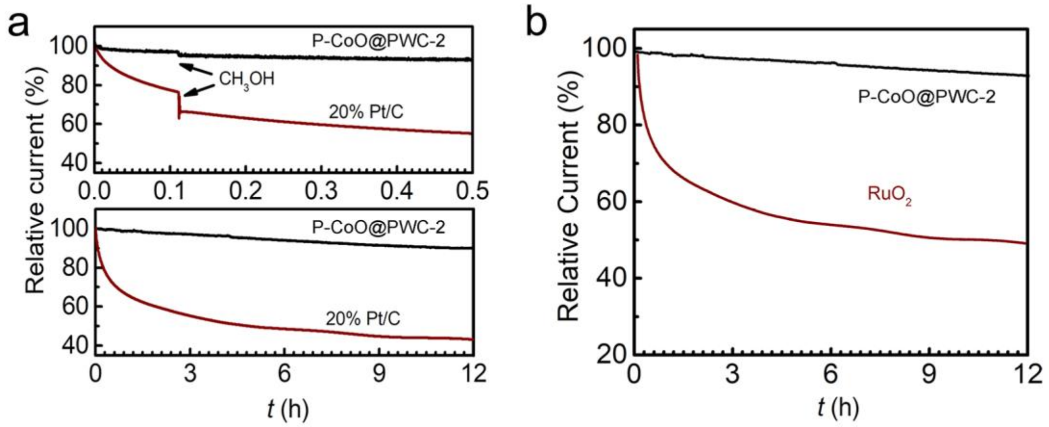

Figure S10. The (a) *i*–*t* responses of P–CoO@PWC–2 and 20 % Pt/C before and after the addition of CH_3OH (top half part), *i*–*t* responses of P–CoO@PWC–2 and 20 % Pt/C for ORR in O_2–saturated KOH (lower half part). The (b) *i*–*t* responses of P–CoO@PWC–2 and RuO_2 for OER in N_2–saturated KOH.

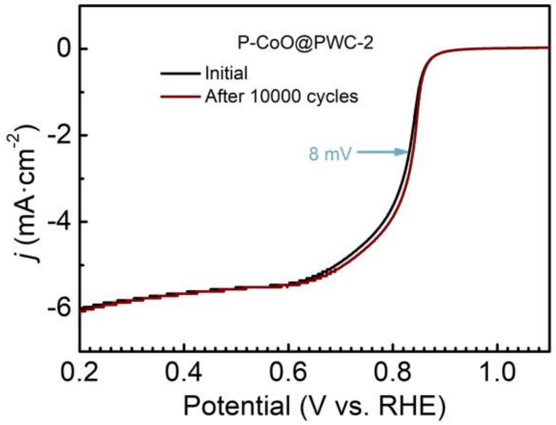

Figure S11. Accelerated durability test (ADT) of P–CoO@PWC–2 in O_2–saturated 0.1 M KOH.

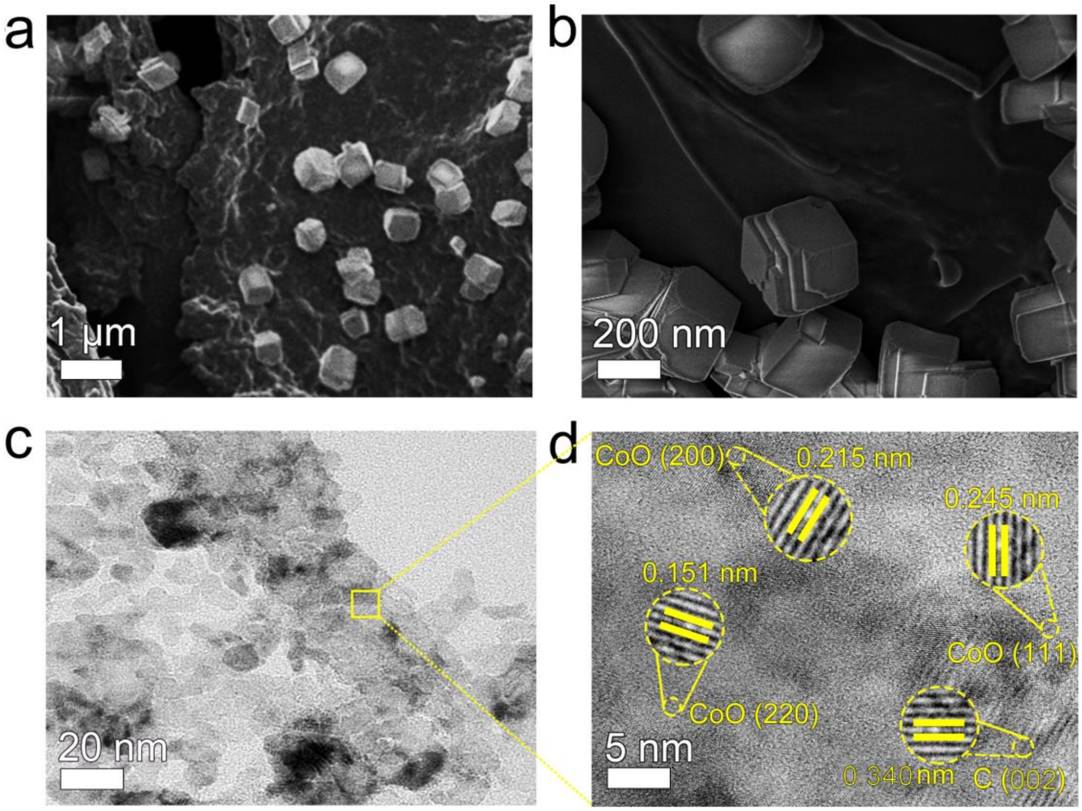

Figure S12. Characterization after durability tests. (a, b) SEM, (c) TEM and (d) HRTEM images of P–CoO@PWC–2.

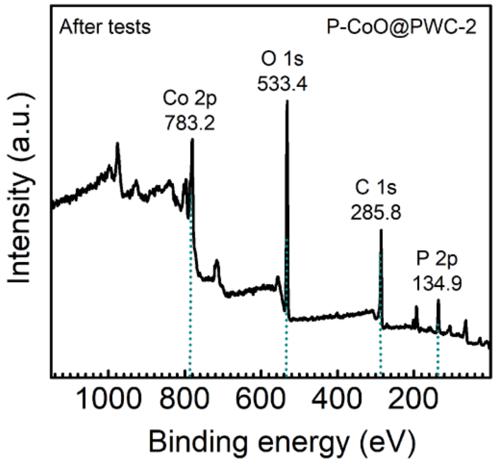

Figure S13. XPS survey spectra of P–CoO@PWC–2 after durability tests.

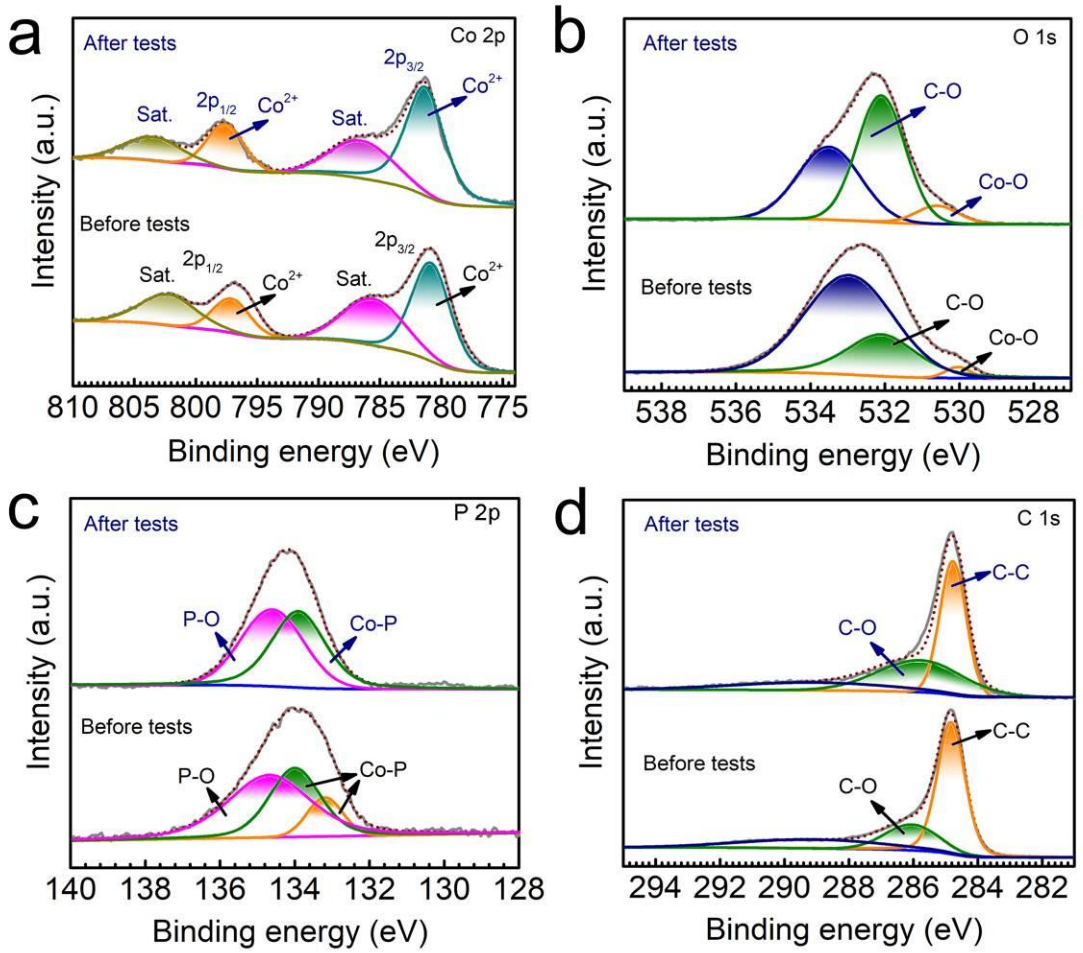

Figure S14. XPS spectra of (a) Co 2p, (b) O 1s, (c) P 2p and (d) C 1s sites in P–CoO@PWC–2 before and after the stability test.

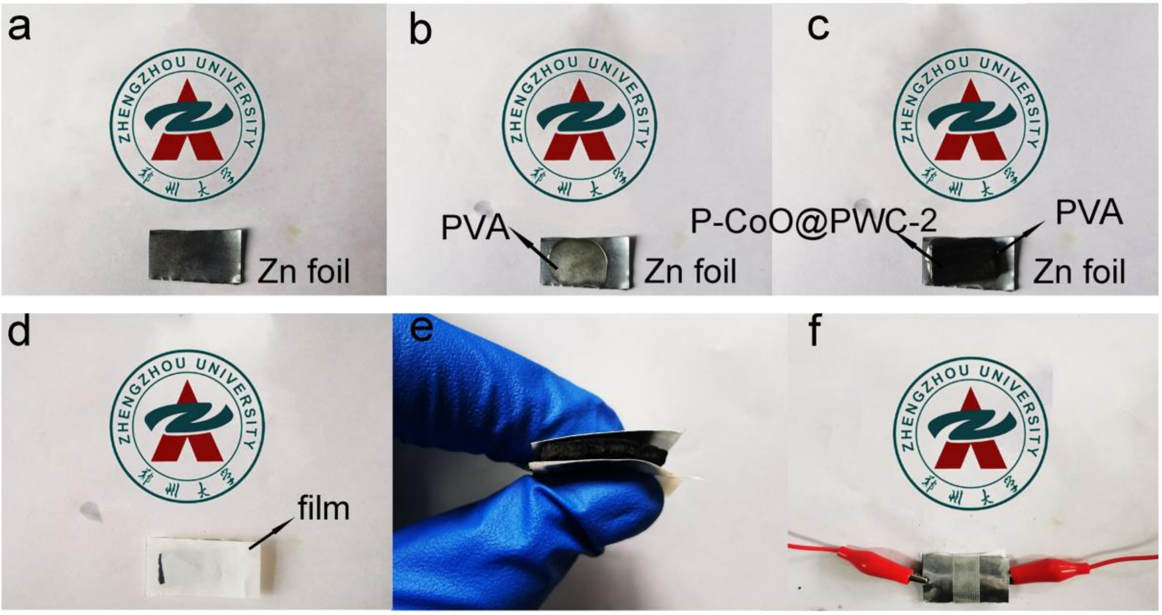

Figure S15. The assemble process (a–d) of P–CoO@PWC–2–based quasi-solid-state rechargeable ZAB (e–f).

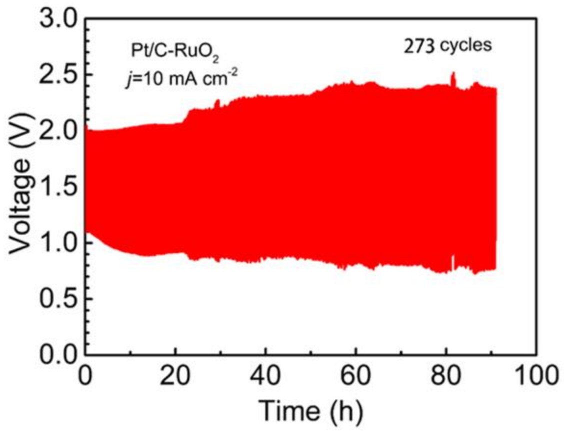

Figure S16. Long–term cycling stability of RZABs based on Pt/C–RuO_2 catalyst.

Reference

[1] J. Yang, L. Chang, H. Guo, J. C. Sun, J. Y. Xu, Y. N. Zhang, Z. M. Wang, L. P. Wang, F. Hao and X. B. Niu, *J Mater. Chem. A* **2020**, *8*, 1229.

[2] X. M. Guo, C. Qian, X. H. Wan, W. Zhang, H. W. Zhu, J. H. Zhang, H. X. Yang, S. L. Lin, Q. H. Kong and T. X. Fan, *Nanoscale* **2020,** *12*, 4374.

[3] W. Jin, J. P. Chen, B. Liu, J. G. Hu, Z. X. Wu, W. Q. Cai and G. T. Fu, *Small* **2019**, *30*, 1904210.

[4] Z. –J. Jiang and Z. Q. Jiang, *Sci. Rep.* **2016**, *6*, 27081.

[5] S. N. Hu, Y. Tan, C. Q. Feng, S. Q. Wang, Z. G. Sun, H. M. Wu and G. X. Zhang, *J. Solid State Electrochem.* **2019**, *23*, 2291.

[6] S. C. Chou, K. C. Tso, Y. C. Hsieh, B. Y. Sun, J. F. Lee and P. W. Wu, *Materials* **2020**, *6*, 2703.

[7] L. Z. Qiu, X. P. Han, Q. Lu, J. Zhao, Y. Wang, Z. L. Chen, C. Zhong, W. B. Hu and Y.D. Deng, *Inorg. Chem. Front.* **2019**, *6*, 3554.

[8] M. Zhu, J. Nong, P. Xie, A. S. Zhu, M. Z. Rong and M. Q. Zhang, *Electrochim. Acta* **2019**, *295*, 624.

[9] L. S. Zhou, B. L. Deng, Z. Q. Jiang and Z. –J. Jiang, *Chem. Commun.* **2019**, *55*, 525.

[10] C. C. Weng, J. T. Ren, Z. P. Hu and Z. Y. Yuan, *ACS Sustainable Chem. Eng.* **2018**, *6*, 15811.

[11] X. B. Huang, J. Q. Wang, H. L. Bao, X. K. Zhang and Y. M. Huang, *ACS Appl. Mater. Interfaces* **2018**, *10*, 7180.
